# The National Association of Dental Examiners

**Published:** 1888-11

**Authors:** 


PROCEEDINGS OF

### THE NATIONAL ASSOCIATION OF DENTAL EXAMINERS.

AT THEIR SEVENTH ANNUAL MEETING HELD IN LOUISVILLE, KY., AUGUST 27TH, 28TH AND 29TH, 1888.

The Seventh Annual Meeting of the National Association of Dental Examiners was called to order by the president, Dr. Geo. H. Cushing, at the Galt House, Louisville, Ky., August 27th, 1888, at 8 P. M.

Dr. Fred A. Levy, Secretary.

Dr. Cushing explained that as he was no longer a member of the Illinois State Board of Examiners, he could not be considered a member of the National Association, so vacated the chair in favor of the Vice-President, Dr. T. S. Waters.

Roll call showed the following State Boards represented :

Illinois, Drs. C. R. E. Koch and R. N. Lawrance ; Georgia, Dr. S. B. Barfield ; New Jersey, Dr. Fred A. Levy ; Mississippi, Dr. W. W. Westmoreland ; South Carolina, Dr. G. F. Wright ; Ohio, Drs. J. Taft and H. A. Smith ; Indiana, Drs. P. G. C. Hunt and S. T. Kirk ; Maryland, Dr. T. S. Waters ; Kentucky, Dr. A. O. Rawls ; Wisconsin, Dr. B. G. Macreklein.

The following letter from the Wisconsin State Board was then read and placed on file :

STATE BOARD OF DENTAL EXAMINERS, WISCONSIN.

LA CROSSE, AUGUST 20TH, 1888.

FRED A. LEVY, D. D. S.,

*Secretary National Association of Dental Examiners.*

DEAR SIR :

It is not positively known that any number of this Board will be present at the coming meeting of the Association at Louisville. Permit us, however, to respond present upon the roll call in sentiment if not in person. There are one or two matters which it seems advisable to bring before the Association, the most important of which has bothered us, viz.: The licensing of first course college students. The practice does not seem right, although they correctly answer the required percentage of questions submitted at their examination. The average standard of qualification for graduation at our colleges is low enough, and for a State Board to license a person who has acquired but the half or even less preparation demanded by the college, seems to represent it as being satisfied with an inferior standard, and willing to admit into practice men of inferior capabilities or less preparation. Doubtless all State laws providing for examinations in lieu of a diploma may be considered weak in their conception of competency or in the disposition to enforce the law in the interest of a higher degree of excellence, and until public sentiment shall have been moulded to permit our laws to be changed, making the diplomas the only basis of qualification Some uniform method might be recommended to assist us and others to overcome this doubtful practice.

2. State Boards cannot be expected to meet oftener than once in every six months. If there is any provision in any State enactment which provides for "temporary licenses," we have not discovered them. So that there is no authority for granting the privilege of engaging in practice in the interim, which is sometimes a source of inconvenience and loss of applicants. If the Association or representatives of any Board can suggest the right course to pursue in this matter such advice will be very acceptable.

3. Is it not desirable to have a better and more uniform list of questions to govern State Boards in examina ions? The Association can improve upon their former efforts in this direction by the appointment of a committee to formulate such list to be submitted at the next annual meeting.

The enclosed copy of our third annual report will show the statements regarding the workings under our law which we regard as one of the best enacted. All members of the profession in the State seem to be well satisfied with its provisions; the Board is harmonious and in earnest in entering it, and extends greeting to all who are laboring to accomplish the same objects.

Respectfully Submitted,

CHARLES C. CHETTENDEN, President.

EDGAR PALMER, Secretary.

On motion a Business Committee of three was appointed, and the communication referred to them. The chair appointed Drs. Taft, Koch and Barfield the Committee. Dr. Koch asked for information as to the standing of the Dental Department of the Howard University; also Dental Department of the National University, both of Washington. On motion the following resolution was adopted:

*Resolved,* that the Secretary be empowered and requested to have printed five hundred copies of the constitution, by-laws, and standing resolutions. Also the following:

*Resolved,* that a Committee of three be appointed to prepare a list of the Dental Colleges which this Association will recommend to the various State Boards whose diplomas may be accepted instead of an examination.

Carried. President appointed Drs. Levy, Rawls and Wright. The meeting then adjourned.

Aug. 28th, meeting called to order. Dr. Waters in the chair; Dr. Levy, Secretary. Minutes were read and approved. Delegates Dr. M. C. Marshall and L. G. Roberts, from the Arkansas State

Board, were present and joined the Association. Business Committee reported as follows :

Your Committee to whom was referred the letter of Dr. Edgar Palmer, Secretary of the Wisconsin Board of Examiners, respectfully recommended the discontinuance of the practice of giving permits to practice dentistry to students during the time of their college work or before their graduation.

We also recommend that any applicant for examination and license to practice dentistry between the regular sessions of the respective State Boards may be examined during such interval by one or more members of a State Board as may be designated by said Board, and upon such examination being satisfactory, a permit may be issued to the applicant to practice dentistry till the next meeting of the Board and no longer. This examination and permit shall in no case exempt the candidate from an examination by the full Board at its next regular meeting. Your Committee does not believe it advisable to have a uniform list of questions for examination throughout the country. We would recommend, however, that State Boards embrace in their examinations the following branches, and that there be not less than ten questions on each of these branches, viz.: Anatomy, physiology, pathology, histology, hygiene, materia medica, therapeutics, chemistry, metallurgy, operative dentistry, prosthetic dentistry and dental jurisprudence. We suggest that each State Board formulate its own list of questions, and that this list be changed at least once each year, and that a standard of at least seventy-five per cent. of correct answers be required.

J. Taft,<br>

Chas. R. E. Koch, } Committee.<br>

S. B. Barfield,

Received and adopted. The Committee on List of Colleges offered a report which was accepted. Dr. Koch resigned from Business Committee on account of having to leave the city, and Dr. Lawrence was appointed in his place. The following resolutions were then offered :

*Resolved*, that the resolution appearing upon the records of this Board, the same being presented in writing by Dr. Coyle and unanimously adopted by this Board at its meeting at New Orleans, the said resolution being in effect to prevent members of faculties from acting as members of State Boards of Dental Examiners, be hereby rescinded.

Carried.

*Resolved*, that a Committee of three be appointed, whose duty it shall be to co-operate, so far as they can, with the profession in the different States in securing a uniformity in the laws regulating the practice of dentistry in the States.

Carried. President appointed Drs. Taft, Rawls and Barfield.

*Resolved*, that the State Boards be requested to furnish the Secretary of the National Association of Dental Examiners with a certified copy of the laws in force and any amendments in their respective States and their efficiency.

Carried. Adjourned.

August 29th; meeting called to order. Dr. Waters in the chair; Dr. Levy, Secretary. Roll call showed the following States represented: Illinois, Ohio, Georgia, Indiana, New Jersey, Maryland, South Carolina, Wisconsin, Kentucky and Arkansas. Minutes of last meeting read and approved on motion. The list of colleges presented and accepted at the last meeting was reconsidered. The list was then referred back to the Committee to be revised, and was again presented to the Association; was amended and accepted, as follows:

1. Baltimore College of Dental Surgery, Baltimore, Md.
2. Boston Dental College, Boston, Mass.
3. Chicago College of Dental Surgery, Chicago, Ill.
4. Harvard University, Dental Department, Cambridge, Mass.
5. Kansas City Dental College, Kansas City, Mo.
6. Minnesota Hospital, Dental Department, Minneapolis, Minn.
7. Missouri Dental College, St. Louis, Mo.
8. New York College of Dentistry, New York City.
9. Ohio College of Dental Surgery, Cincinnati, O.
10. Pennsylvania College of Dental Surgery, Philadelphia, Pa.
11. Philadelphia Dental College, Philadelphia, Pa.
12. St. Paul Medical College, Dental Department, St. Paul, Minn.
13. University of California, Dental Department, San Francisco, Cal.
14. University of Iowa, Dental Department, Iowa City, Ia.
15. University of Michigan, Dental Department, Ann Arbor, Mich.
16. University of Pennsylvania, Dental Department, Philadelphia, Pa.
17. Vanderbilt University, Dental Department, Nashville, Tenn.
18. Northwestern College of Dental Surgery, Chicago, Ill.
19. Louisville College of Dentistry, Louisville, Ky.
20. Indiana Dental College, Indianapolis, Ind.
21. Dental Department of Northwestern University, Chicago, Ill.
22. Dental Department of Southern Medical College, Atlanta, Ga.
23. Dental Department of University Tennessee, Nashville, Tenn.
24. School of Dentistry of Meharry Medical Department of Central Tennessee College, Nashville, Tenn.

The Association then proceeded to the election of officers with the following result: President, Dr. T. S. Waters, of Baltimore, Md.; Vice-President, Dr. S. T. Kirk, Kokomo, Ind.; Secretary and Treasurer, Dr. Fred A. Levy, Orange, N. J.

On motion it was resolved that when we adjourn we do so to meet on the day and at the place of meeting of the American Dental Association, at 9.30 A. M. (*I. e.* Saratoga, Tuesday, August 5th, 1889). Minutes were then read and approved and the meeting adjourned. FRED A. LEVY, Secretary.

